# Effectiveness of the dog therapy for patients with dementia - a systematic review

**DOI:** 10.1186/s12888-019-2245-x

**Published:** 2019-09-06

**Authors:** Blanka Klimova, Josef Toman, Kamil Kuca

**Affiliations:** 1 Department of Applied Linguistics, Faculty of Informatics and Management, University of Hradec Kralove, Hradec Kralove, Czech Republic; 2 Biomedical Research Centre, University Hospital Hradec Kralove, Hradec Kralove, Czech Republic; 3 Centre for Basic and Applied Research, Faculty of Informatics and Management, University of Hradec Kralove, Sokolska 581, Hradec Kralove, Czech Republic

**Keywords:** Dogs, Dementia, Therapy, Intervention, Effectiveness, Review

## Abstract

**Background:**

Dementia represents a mental and economic burden for both patients and their caregivers. Therefore, the aim of this study is to explore the effectiveness of animal assisted therapy (AAT) with special focus on canis therapy among people with dementia, specifically Alzheimer’s disease.

**Methods:**

The key method of this review study is a systematic review of the research studies detected in the Web of Science, Scopus and PubMed. The search was conducted for the studies dating from 2016 till 31 August 2018 because several review studies were published before. Eventually, only six studies were involved into the final analysis.

**Results:**

The findings of this review, based on significant effect sizes, reveal that AAT may work as a beneficial and effective complementary treatment, especially in the area of behavioral and psychological symptoms, for patients with different degree of dementia severity if AAT is targeted at their specific needs and interests.

**Conclusions:**

More research in the area of methodology for the implementation of AAT is necessary, and more research should be conducted with respect to the use of AAT for the improvement of cognitive functions in people with dementia.

## Background

Nowadays, the number of people suffering from dementia worldwide, particularly Alzheimer’s disease, reaches about 50 million. It is estimated that every year there occur about 10 million new cases [1]. Dementia is a neurodegenerative syndrome, which causes deterioration of cognitive functions, especially thinking, orientation, memory, or communication. The cognitive impairment is usually accompanied with other symptoms such as behavioral disorders, difficulties in walking, sleeping, or sexual problems [2].

Dementia results from different diseases, for example, strokes, malnutrition, or brain tumors [3]. Alzheimer’s disease (AD) seems to be the most common form of dementia and contributes to 70% of dementia cases. The other most frequent types of dementia are vascular dementia, dementia with Lewy bodies, Parkinson’s disease dementia, frontotemporal dementia/ degeneration, and mixed dementia [4]. Dementia usually starts to affect people at the age of 60+ years. But, for example, frontotemporal dementia occurs as early as at the age of 45 [5]. At present, dementia symptoms cannot be cured and inevitably lead to patient’s disability and eventually, to his/her death. Depression and cognitive decline especially result in patient’s mortality among the elderly people with dementia [6, 7]. Adequate medications can for some time postpone this process. Nevertheless, the symptoms of dementia steadily deteriorate and the patient cannot look after himself/ herself. S/he has to rely on somebody else in this respect, most often on his/her family member. For instance, in the year of 2016, about 16 million of informal carers delivered 18 billion hours of care [8]. This care inevitably impacts these informal carers, especially physically, emotionally, but also financially since they usually must quit their job to take care of their loved ones [9]. Therefore, dementia significantly influences not only patients, but also their carers [10]. For this reason, scientists worldwide are seeking non-pharmacological strategies that can enhance or maintain cognitive functions and psychical symptoms of these people in order to help them in maintaining quality of their life and reducing the overall economic burden. These alternative approaches are non-invasive, with minimum side effects and definitely less costly [11]. One such non-pharmacological therapy is animal assisted therapy.

Animal-assisted therapy (AAT) can be defined as *a goal directed intervention in which an animal meeting specific criteria is an integral part of the treatment process* [12]. AAT is usually performed by health or human service providers. These are, for instance, registered nurses, nursing assistants, or occupational therapists [13]. The aim of AAT is to enhance physical, social, emotional, or cognitive functions in both healthy and unhealthy individuals and thus, contribute to the improvement of person’s well-being. AAT can take a form of individual or group interventions [12].

As research [14] shows, AAT improves a person’s mental and physical health. In the area of mental health, it releases an automatic relaxation response, reduces the feeling of anxiety, contributes to the lowering of loneliness, or help in the recall of memories. In the area of physical health, AAT helps to reduce blood pressure and improves cardiovascular health, decreases the amount of medications, reduces physical pain, or helps children with autism to get engaged in social interactions. For individuals with dementia, AAT has the following specific benefits:

- it contributes to slightly higher physical activity; people can pet the animal, such a dog, or in better cases, they can go for a walk [15, 16];
- it can relieve the so-called sundown syndrome, which manifests itself in increased agitation, restlessness, disorientation and aggressive behavior [15, 17];
- it can improve short-term memory and communication skills [15, 18];
- it enhances eating habits [16];
- it reduces loneliness [15, 17, 18].

In fact, when patients pet or cuddle their animal, their body releases endorphins and other hormones such as oxytocin, prolactin and dopamine. This contributes to the benefits described above [19]. As Jo [19] indicates, the most suitable animals for ATT are fish, cats, dogs and horses. Most recently, robots disguised as animals also started to be used in ATT [20]. They appear to have similar positive effects without the negative aspects of traditional pets such as allergies, infections, or biting [21]. As Petersen et al. [21] demonstrated in their study, these animal robots can reduce stress and anxiety in people with dementia and decrease the use of psychoactive medications and pain medications among them.

Although several review studies [22–25] had been already published, this review includes the latest studies on this topic, which were not included in these reviews with one exception in [25]. In addition, this review apart from the effect of canis therapy on the reduction of behavioral symptoms of dementia explores also its effect on the reduction of cognitive symptoms of dementia. In this respect it differs from the latest systematic review by Yakimicki et al. [26] who did not concentrate on the relationship between the cognitive symptoms of dementia and animal-assisted interventions.

Thus, the purpose of this study is to systematically review evidence from controlled trials, case reports, observational or experimental studies in order to find out the answer to the question whether animal assisted therapy with a special focus on canis therapy among people with dementia is effective in reducing cognitive and behavioral symptoms of dementia.

## Methods

The authors performed a systematic review of research studies detected in the Web of Science, Scopus and PubMed. The search keywords were as follows: *animal assisted therapy* AND *dementia*, *animal assisted therapy* AND *Alzheimer’s disease, canis therapy* AND *dementia, canis therapy* AND *Alzheimer’s disease, dog therapy* AND *dementia, dog therapy* AND *Alzheimer’s disease*. The search was conducted for the studies dated from 2016 till 31 August 2018.

Two (BK and JT) of the authors identified and screened the relevant articles. Altogether 107 articles were detected in the databases mentioned above. Most of the studies were detected in Web of Science (62), followed by Scopus (30), and PubMed (15). After a thorough review of the titles and abstracts (53) and their duplication (22) of the selected studies, 32 studies were screened and after that, 21 studies remained for the full-text analysis.

These full-text articles were then analyzed and evaluated on the basis of the following inclusion and exclusion criteria. The inclusion criteria, based on PICOS guidelines, were as follows:

- The articles had to be published between January 1, 2016, and August 31, 2018.
- Only English peer-reviewed journal articles were involved.
- The subjects had to be patients with dementia.
- The intervention had to include a dog therapy.
- Only randomized controlled trials, experimental/cross-sectional studies, case reports, or observational studies were involved.
- The primary outcome focused on the reduction of dementia symptoms, especially cognitive decline among patients with dementia.
  The exclusion criteria were as follows:
- Descriptive or review studies on the research topic were excluded from the analysis.
- Studies whose subjects were not all patients with dementia were also excluded, e.g. [27].
- Studies which did not focus on dog therapy were not involved either, e.g. [21].

In addition, a backward search was also performed, i.e., references of detected studies were evaluated for relevant research studies that authors might have missed during their search. In addition, a google search was conducted in order to detect unpublished (grey) literature. After this, another two articles have been identified. Thus, altogether six research articles were eventually analyzed and evaluated.

Two authors (BK and JT) performed an independent quality assessment of these studies. They read the articles to assess eligibility and to determine the quality. The basic quality criteria were adequately described study design, participant characteristics, control conditions, outcome measures, and key findings, with special focus on statistically significant differences (Table 1). The authors selected these basic quality criteria using Health Evidence Quality Assessment Tool for review articles [31].

**Fig. 1:**
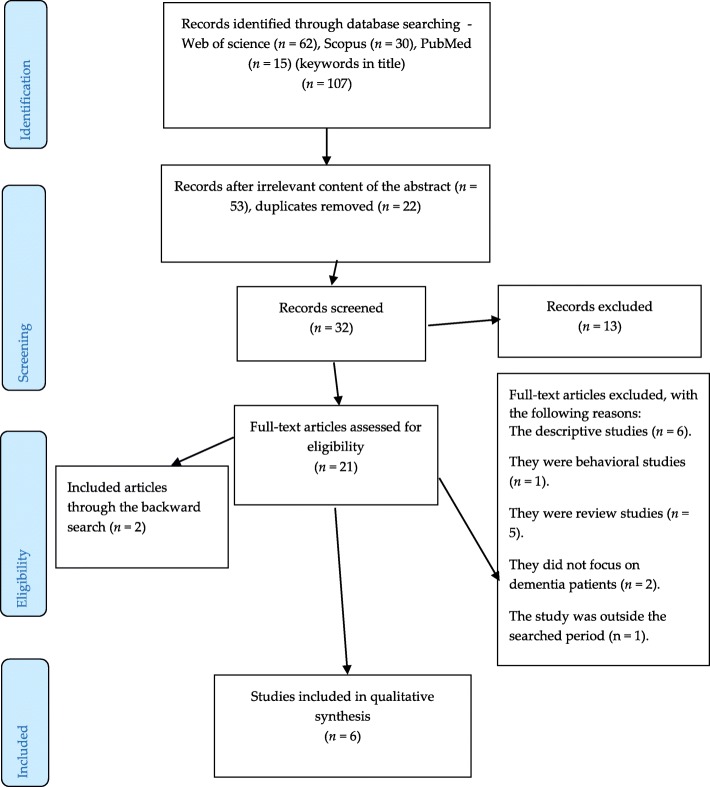
An overview of the selection procedure

Figure 1 below then describes the selection procedure of the detected studies.

## Results

Altogether six original studies on the research topic were identified. Three studies were controlled studies [28–30], two studies were experimental studies [17, 18] and one study was an observational study [13]. As for the country of origin, most of the research in this area has been conducted in Scandinavian countries [13, 17, 29, 30], followed by Italy [28] and USA [18]. The key area of assessment focused on behavioral symptoms of patients with all severity of dementia [13, 18, 29, 30]. Apart from that, one study [28] explored cognitive functions and one study [17] concentrated on physical health and more specifically, on balance. The sample of subjects ranged from 5 to 80 older individuals with the mean age between 75 to 95 years. Not surprisingly, there were more female participants than male participants if taking into account their age. The intervention period lasted from two weeks to six months. All control groups were passive. The main outcome measures comprised standardized tests such as Mini-Mental State Examination, Geriatric Depression Scale, Clinical Dementia Rating, or Quality of Life in Late-stage Dementia. The results of all identified studies indicate that the AAT or AAA therapies with a dog have a positive impact on mental and physical health of the subjects with dementia as the effect sizes show. Based on the basic quality criteria, only the study by Swall et al. [13] seems to be less reliable quality. The findings of the detected studies are summarized in alphabetical order of their first author in Table 1 below.

**Table 1:** An overview of the findings from the selected studies

Study	Characteristics of subjects	Description of intervention (type, period)	Assessment areas	Outcome measures	Results, statistically significant differences
Menna et al. [28]	50 individuals with mild-to-moderate AD, mean age 75 years, 37 females, 13 males.	3 groups – 2 intervention groups- reality orientation therapy (ROT) and AAT (20 subjects in each group) and 1 control group with no stimulation; once a week for 45 min for 6 months.	Cognitive skills (attention, language skills, spatial-temporal orientation), emotional aspects such as mood.	Mini-Mental State Examination (MMSE), 15-item Geriatric Depression Scale (GDS), Bonferroni-Dunn test.	The findings indicate that both intervention groups improved their emotional status, i.e., the AAT group improved from 11.5 (T0) to 9.5 (T1), and the ROT group improved from 11.6 (T0) to 10.5 (T1) on GDS; and the AAT group also slightly enhanced their cognitive skills, i.e. mean MMSE was 20.2 at T0 and 21.5 at T1.
Olsen et al. [17]	49 subjects, out of which 21 were located in nursing homes and had severe dementia a 28 were home-dwelling with moderate dementia, mean age 84 years.	2 groups with the same group dog intervention therapy two times a week for 30 min for 3 months.	Behavioral aspects.	MMSE, Clinical Dementia Rating (CDR), video recordings.	The results show that the therapy has a positive impact on mental health I both groups; however, with smaller intensity among patients in the nursing homes due to their severity of dementia.
Olsen et al. [29]	80 subjects with mild-to-moderate dementia; mean age between 82 and 84 years.	1 intervention group with animal-assisted activity (AAA) with a dog and 1 passive control group; it lasted two times a week for 30 min for 3 months.	Physical health and quality of life (QoL).	Berg balance scale, Quality of Life in Late-stage Dementia, CDR.	The findings reveal that the AAA intervention had a significant effect (*p* = 0.03) on improving subjects’ balance in comparison with the control group. However, there was no effect on QoL.
Olsen et al. [30]	58 subjects with dementia; mean age between 83 and 84 years.	1 intervention group with animal-assisted activity (AAA) with a dog and 1 passive control group; it lasted two times a week for 30 min for 3 months.	Neuropsychiatric symptoms and QoL.	MMSE, Cornell Scale for Depression in Dementia, Brief Agitation Rating Scale, Quality of Life in Late-stage Dementia, CDR.	A significant effect on depression (*p* = 0.001) and QoL (*p* = 0.003) was detected for participants with severe dementia at follow-up after 3 months. For QoL, a significant effect of AAA was also found immediately after the intervention. No effects on agitation and cognitive functions were detected.
Pope et al. [18]	44 subjects with primary dementia; mean age: 79.8 years.	1 group had AAT intervention and 1 group human interactions (reading and conversation). It was held twice a week for 10 min for 2 weeks.	Social behavior.	Demographic and Pet History Questionnaire, Social Behaviors Checklist, Menorah Park Engagement Scale, Cohen-Mansfield Agitation Inventory.	The results show that both interventions had a positive impact on the enhancement of behavioral scores (*p* < .001).
Swall et al. [13]	5 subjects with AD, aged between 89 and 95 years, 4 women and 1 man.	AAT, 50 sessions.	Behavioral symptoms.	Video recordings, phenomenological hermeneutics.	The findings of this study indicate that the dog therapy had a positive effect on patients] behavior in the area of reduced agitation and enhancement of the feeling of empathy and affection.

## Discussion

As the findings of the studies in Table 1 show, AAT or AAA therapies may be effective in the care about patients with dementia. They especially positively enhance patients’ behavior since while being with a dog, patients appear to be calm, relaxed and contented, which results in the reduction of their feelings of depression, anxiety, agitation, and aggression [13, 17, 18, 28, 30]. This was also supported by significant effect sizes [17, 18, 28]. In addition, AAT/AAA contributes to the improvement of social behavior; the presence of a dog stimulates patients to interact and thus reduces their social isolation and loneliness. These findings have been also confirmed by other review studies, e.g. [22, 23, 25, 26], and in most previous empirical studies, e.g. [32, 33]. The repeated multimodal stimulations (verbal, visual, tactile) as seen in the study by Menna et al. [28] prove to be feasible and effective. In this study, the intervention took the form of structured play with a dog, which acted as a therapeutic and social agent.

Furthermore, dog therapy can be effective in the improvement of patients’ physical health as it was proved in the study on balance by Olsen et al. [29], in which AAA had a significant effect (*p* = 0.03) on improving subjects’ balance and preventing risks of fall in comparison with the control group. The researchers engaged patients in active interactions with the dog and the patients, for example, had to bend down to pick the ball or they leaned forward to pet the dog. All these movements required a good posture control. Positive outcomes in the area of physical health were also studied by Cherniack and Cherniack [24], but with modest results.

In addition, dog therapy may also be beneficial for the improvement of cognitive functions as it was demonstrated by Menna et al. [28], although with modest effects. Interaction with the dog namely requires from the patient to pay attention, orientation in his/her environment, or simply it evokes in him/her memories from the past.

Olsen et al. [17] in their study also point out that the severity of dementia should be considered when planning AAT/AAA since patients with severe dementia had different needs and they demand more care and time. Therefore, AAT/AAA should be tailored to their specific needs and interests and aim at the person-centered dementia care [13].

The limitation of this study consists in analyzing the results of studies with different methodological approaches to AAT or AAA, small subject samples, as well as with different intervention periods. In addition, there was only one study [30], which also measured the effect after the follow up period. All these aspects then might have an impact on the overestimation of the discussed findings. Therefore, standard guidelines for the implementation of AAT are needed [22].

## Conclusion

The findings of this study reveal that AAT may work as a beneficial and effective complementary treatment (especially in the area of behavioral and psychological symptoms) for patients with different degree of dementia severity if AAT is targeted at their specific needs and interests. Nevertheless, more research in the area of methodology for the implementation of AAT is necessary, as well as more research should be conducted with respect to the use of AAT for the improvement of cognitive functions in people with dementia.

## Data Availability

The search strategies used in this systematic review have been included in the Methods and all of the manuscripts informing this systematic review are listed in Table 1.

## Abbreviations

AAA: animal assisted activity
AAT: animal assisted therapy
AD: Alzheimer’s disease
CDR: Clinical Dementia Rating
GDS: Geriatric Depression Scale
MMSE: Mini-mental State Examination
QoL: Quality of Life
ROT: reality orientation therapy
